# Enablers and barriers to vaccine uptake and handwashing practices to prevent and control COVID-19 in Kenya, Uganda, and Tanzania: a systematic review

**DOI:** 10.3389/fpubh.2024.1352787

**Published:** 2024-03-27

**Authors:** Josphat Martin Muchangi, James Mturi, Hajra Mukasa, Kioko Kithuki, Sarah Jebet Kosgei, Lennah Muhoja Kanyangi, Rogers Moraro, Maureen Nankanja

**Affiliations:** ^1^Amref Health Africa, Nairobi, Kenya; ^2^Amref Health Africa, Dar es Salaam, Tanzania; ^3^Amref Health Africa, Kampala, Uganda

**Keywords:** hesitancy, handwashing, hand hygiene, acceptancy, uptake, COVID-19 vaccine, barriers, enablers

## Abstract

The global emergence of coronavirus disease 2019 (COVID-19) posed unprecedented challenges, jeopardizing decades of progress in healthcare systems, education, and poverty eradication. While proven interventions such as handwashing and mass vaccination offer effective means of curbing COVID-19 spread, their uptake remains low, potentially undermining future pandemic control efforts. This systematic review synthesized available evidence of the factors influencing vaccine uptake and handwashing practices in Kenya, Uganda, and Tanzania in the context of COVID-19 prevention and control. We conducted an extensive literature search across PubMed, Science Direct, and Google Scholar databases following Preferred Reporting Items for Systematic Review and Meta-Analysis (PRISMA) guidelines. Out of 391 reviewed articles, 18 were eligible for inclusion. Some of the common barriers to handwashing in Kenya, Uganda, and Tanzania included lack of trust in the government’s recommendations or messaging on the benefits of hand hygiene and lack of access to water, while some of the barriers to vaccine uptake included vaccine safety and efficacy concerns and inadequate awareness of vaccination sites and vaccine types. Enablers of handwashing practices encompassed hand hygiene programs and access to soap and water while those of COVID-19 vaccine uptake included improved access to vaccine knowledge and, socio-economic factors like a higher level of education. This review underscores the pivotal role of addressing these barriers while capitalizing on enablers to promote vaccination and handwashing practices. Stakeholders should employ awareness campaigns and community engagement, ensure vaccine and hygiene resources’ accessibility, and leverage socio-economic incentives for effective COVID-19 prevention and control.

**Clinical trial registration**: [https://clinicaltrials.gov/], identifier [CRD42023396303].

## Introduction

The Coronavirus disease 2019 (COVID-19) has brought about unprecedented challenges globally, threatening to undo the decades of progress in health systems, education and poverty eradication (1). To date, the pandemic has resulted in more than 6.9 million deaths, loss of jobs equivalent to 114 million, and about half of 3.3 billion workforce is on the verge of losing their livelihoods globally, thus raising concern regarding the appropriate responses (2, 3).

Countries around the globe have taken measures such as mass vaccination, wearing of masks, and setting up vaccination centers and handwashing stations in public places to control the pandemic (4–7). Whilst there has been a significant decline in new infections, a vaccine against COVID-19 is widely viewed as the safest health strategy to protect the public against transmission of severe acute respiratory syndrome coronavirus 2 (SARS-CoV-2) (8, 9). The COVID-19 vaccines have been associated with a tremendous efficacy ranging between 50 to 90% and almost 100% protection from loss of life where one article determined the vaccine efficacy based on the prevention of symptomatic laboratory-confirmed COVID-19 in individuals without evidence of previous SARS-CoV-2 infection (9) while another determined the vaccine efficacy based on the reduction in the risk of COVID-19 cases among individuals who received the vaccine compared to those who received a placebo (8). As such, considerable investments in the manufacturing and rollout of these vaccines for emergency use have added a huge boost to the fight against COVID-19.

Equally, hand hygiene practices such as handwashing continue to be a critical strategy in reducing the transmission of SARS-CoV-2 and other related respiratory viruses. The World Health Organization (WHO) notes that handwashing with soap and water is the most effective low-cost strategy to minimize the transmission of SARS-CoV-2. Studies have shown that hand hygiene, when used effectively, can decrease transmission of other respiratory infections during the COVID-19 pandemic (10).

Given the importance of COVID-19 vaccines and handwashing, several studies have been carried out to determine the barriers and enablers to the uptake of vaccines and handwashing. In a study to assess hand hygiene compliance in India, it was indicated that spreading out facilities with clean water, soap, basins, and hand rub enhanced handwashing practices (10). Further, an assessment of hand hygiene practices among health care workers in Riyadh, Saudi Arabia, showed that the participants who had formal hand hygiene instruction used it regularly throughout the COVID-19 pandemic (11). Investigation of the obstacles to hand hygiene practices in sub-Saharan Africa revealed that to improve hand hygiene practices, it was necessary to offer education on the topic and sustainable solutions to the water shortage, disinfectants, and incentives, among other remedies (12). In addition to synthesizing factors influencing vaccine uptake and handwashing practices in Kenya, Tanzania, and Uganda, it is essential to acknowledge the diverse socio-economic landscapes, particularly in rural areas. Internet access in these regions plays a crucial role in disseminating health information, including guidance on vaccination and hygiene practices. The current situation in rural areas varies, with challenges such as limited access to social media information (13).

Evidence-based tactics in overcoming vaccine reluctance among Americans revealed that partnership between policymakers and the community was crucial in reducing vaccination hesitancy (14). The usage of public health surveillance systems to collect and process data could provide timely and accurate health information for dissemination and decision-making, such as vaccine distribution (14). In addition, community participation in vaccine distribution boosts vaccine uptake in an African setting, depicting the important role of cross-sector cooperation in helping people to access vaccines (15).

On the contrary, lack of trust in COVID-19 vaccines has resulted in vaccine reluctance (14). Despite the prior development of mRNA-based vaccines for relatively niche diseases, their application on a massive scale during the COVID-19 pandemic marked a paradigm shift. The relatively swift deployment of this advanced technology, coupled with the unprecedented challenges posed by intensive and uncontrolled internet coverage, introduced new complexities. The use of mRNA-based vaccines, previously reserved for specific diseases, now faced both accelerated implementation and widespread dissemination of information, including misinformation. Other causes of low uptake of COVID-19 vaccines include fear of needles or blood, safety worries and religious philosophical convictions. Most of these factors resulted from false information and misunderstandings (16). Despite several studies examining the enablers and barriers to COVID-19 vaccination and hand hygiene practices in different countries, the uptake of COVID-19 vaccines and handwashing practices is low in some countries. This situation threatens to undermine the future successes of immunization campaigns. This can be a challenge in building herd immunity. Handwashing habits and the East African region have received noticeably less attention in reviews that have been published thus far, which have mostly concentrated on factors impacting the intention and uptake of COVID-19 vaccines on the African Continent (17). The urgent need to recognize, understand, and deal with the distinct social, economic, and healthcare contexts that exist in Kenya, Uganda, and Tanzania is what motivated this comprehensive study. These environments have a significant impact on health-related behaviors, especially vaccination uptake and handwashing habits. The complex interactions between various socio-cultural elements, such as religious convictions, community institutions, and cultural traditions, shape how the public views and accepts health initiatives. Economic factors influence people’s ability to take preventive health practices and contribute to varying access to healthcare resources. Vaccine availability and the spread of health information are highly dependent on the state of healthcare systems, from infrastructure to health education programs. Furthermore, the distinct epidemiological terrain, political intricacies, and geographical heterogeneity introduce further facets of intricacy to health-associated decision-making within these areas. Therefore, developing targeted and culturally aware public health initiatives that can successfully promote immunization and hygiene behaviors within each nation requires an in-depth comprehension of these complex circumstances. Therefore, this review aimed at bridging the existing knowledge gaps, guiding the development of tailored public health interventions, and advancing the global understanding by offering context-specific insights by systematically reviewing and synthesizing existing evidence on the enablers and barriers of vaccine uptake and handwashing practices in the context of COVID-19 in Kenya, Uganda, and Tanzania. Our selection of these three countries as focus areas for the systematic review was deliberate and grounded in practical considerations. Conducting a systematic review focusing on Kenya, Uganda, and Tanzania is justified by the need to comprehensively understand the regional dynamics influencing COVID-19 vaccine uptake and handwashing practices within the East African context. Studies such as those by Nabukeera (18) and Davis et al. (19) emphasize the variability in healthcare systems across regions, highlighting the importance of examining the unique challenges and strengths within the healthcare systems of these specific countries. Furthermore, research by Abubakar et al. (20) and Bakeera et al. (21) underscores the significance of cultural and socio-economic factors in shaping public health behaviors, necessitating an examination of local contexts and cultural nuances. Additionally, studies by Briceno et al. (22) and Muchangi et al. (23) highlight the importance of considering epidemiological heterogeneity when designing interventions, emphasizing the need to assess the specific epidemiological factors influencing vaccine uptake and handwashing practices in this region. Thus, a focused review of Kenya, Uganda, and Tanzania provides valuable insights into addressing the pandemic within the East African context, enabling tailored interventions and policy recommendations to tackle COVID-19 effectively. Furthermore, it informs actionable strategies that contribute significantly to broader preparedness and responses to infectious disease outbreaks in the region.

## Materials and methods

### Reporting guidelines

This systematic review was conducted in accordance with the Preferred Reporting Items for Systematic Review and Meta-Analysis (PRISMA) and the Centre for Reviews and Dissemination (CRD) guidelines (24, 25). The review protocol was registered on the PROSPERO, registration number CRD42023396303 and an updated literature search was conducted in November 2023.

### Inclusion criteria

Our review considered the following categories of studies for inclusion: peer-reviewed primary studies that focused on the enablers of handwashing and vaccination to prevent COVID-19 in Kenya, Uganda, and Tanzania; studies assessing the barriers to handwashing and vaccination to prevent and control COVID-19 in Kenya, Uganda and Tanzania; studies published in English language between December 2019 and January 2023.

### Exclusion criteria

We excluded: preprints, letters, commentaries, reviews, conference abstracts, and case series.

### Literature search

In the context of this study, “vaccine uptake” referred to the acceptance and utilization of COVID-19 vaccines by individuals within the populations of Kenya, Uganda, and Tanzania while “handwashing practices” pertained to the behaviors and routines related to hand hygiene, particularly the act of washing hands with soap and water. We searched PubMed, Science Direct and Google Scholar for published studies which investigated the enablers and barriers to handwashing and vaccination to prevent and control COVID-19 in Kenya, Uganda and Tanzania without date or language limitations. Manual screening of the eligible articles was conducted to identify additional publications. The PubMed search strategy was formulated based on population, exposure, comparators, outcomes (PECO) framework using Medical Subject Heading (MeSH) terms for enablers, barriers, handwashing, vaccine, COVID-19, prevention, Kenya, Uganda, and Tanzania (supplementary Tables S1, S2). This search strategy was modified accordingly to suit other databases.

### Study selection and data extraction

We used Mendeley for reference management of the potentially relevant articles. We first screened the articles by title and abstract after which we reviewed full texts of the publications to determine if they met the inclusion criteria. JM and KK selected the eligible studies and extracted data after which the outcomes were compared and disagreements resolved through discussion with a third reviewer, RM. We extracted the relevant information from the research articles using a predefined and standardized data extraction workbook (Table 1). The extracted variables included: name of the first author(s) and year of publication, the title of the study, country of interest, study objective(s), study design, outcome definition, main findings -enablers and barriers to COVID-19 vaccine uptake and handwashing practice. We defined enablers and barriers as reasons encouraging or restraining the uptake of COVID-19 vaccines and handwashing practice, respectively.

**Table 1:** Studies that reported barriers to COVID-19 vaccine uptake and handwashing practice to prevent and control COVID-19.

References	Title of paper	Country	Study design	Sample size (male)	Participants	Age [mean(sd)]	Dates of data collection	Outcome	Outcome definition	Main findings
Konje et al. (26)	The Coverage and Acceptance Spectrum of COVID-19 Vaccines among Healthcare Professionals in Western Tanzania: What Can We Learn from This Pandemic?	Tanzania	Cross-sectional study	811 (423)	Healthcare professionals of different cadres from health facilities in western Tanzania	35 (9.0) years	13 and 26 September 2021	Barriers to COVID-19 vaccine uptake	Perceived barriers for COVID-19 vaccine uptake and factors associated with hesitancy of COVID-19 vaccine among health professionals	The majority (62%) of participants were in the hesitancy stage due to issues related to lack of effective communication and reliable information regarding efficacy and safety.
Mwai et al. (27)	Assessment of water, sanitation and hygiene practices for prevention and control of COVID-19 in Kenya	Kenya	Cross-sectional survey	612 (181)	Household heads (men and women), residing in Kilifi and Mombasa counties	38.2 (14.8) years	25 November and 3 December 2020,	Barriers to handwashing	Factors that hindered handwashing practices	396 (64.7%) households reported challenges in accessing soap. Topping the list of the challenges was that there are other priorities (62.4%) and soap was too expensive (57.2%).
Muchiri et al. (28)	Unmet need for COVID-19 vaccination coverage in Kenya	Kenya	Mixed methods study	622 vaccination sites	Approved COVID-19 vaccination sites comprising of dispensaries, health centres and hospitals	Not applicable	April–July 2021	Barriers to vaccination coverage	Time taken to travel to the vaccination site as a barrier to COVID-19 vaccination	The probability of being vaccinated generally decreased with increase in mean travel times to the COVID-19 vaccination sites. Additionally, there was a negative association between the vaccination coverage and the proportion of population residing in rural areas with a 27.8% decline.
Kabagenyi et al. (29)	Factors Associated with COVID-19 Vaccine Hesitancy in Uganda: A Population-Based Cross-Sectional Survey	Uganda	Population-Based Cross-Sectional Survey	1,042 (462)	Adults ages 18 and above from rural and urban settings	40 (NA) years	June to November 2021	Barriers to vaccine uptake	Factors reducing vaccine uptake	Participants were hesitant to receive COVID-19 vaccine due to myths and misconceptions about SARS-CoV-2 virus and the vaccine itself. For instance, 15% of the participants believed that the vaccine could cause infertility or the vaccine could infect them by spreading the virus into their bodies.
Ouni et al. (30)	COVID-19 vaccine hesitancy among health workers in rural Uganda: A mixed methods study	Uganda	Mixed methods study	346 (151)	registered and practicing health workers in Dokolo district from both government and private health facilities	31.4 (6.9) years	NA	Vaccine hesitancy	Factors associated with vaccine hesitancy	Factors associated with vaccine hesitancy included fear of side effects (Adjusted Odds Ratio [AOR]: 2.55; 95% Confidence Interval [95%CI]: 1.00, 6.49) and health workers’ lack of trust in the information provided by health authorities (AOR: 6.74; 95% CI: 2.43, 18.72). Similar factors were associated with vaccine hesitancy when we used the vaccine hesitancy score. Fear of side effects, distrust in vaccine stakeholders, and lack of trust in the vaccine were barriers to COVID-19 vaccination among health workers.
Ocholla et al. (31)	Association of Demographic and Occupational Factors with SARS-CoV-2 Vaccine Uptake in Kenya	Kenya	Digital cross-sectional survey	171 (103)	Individuals across 47 counties in Kenya	36–60 years	2nd March and 5th March, 2021.	Hesitancy in vaccine uptake	Unwillingness to be inoculated	Out of those who were unwilling to be inoculated, the majority alleged concerns on the side effects.
(32)	The critical need for WASH in emergency preparedness in health settings, the case of COVID-19 pandemic in Kisumu Kenya	Kenya	Qualitative case study design	15 (10)	County government officials and eight were NGO officials	NA	August and September 2020	Handwashing practices	Level of preparedness of accessing handwashing	All participants indicated the healthcare system was ill-prepared for the pandemic making healthcare workers to experience severe psychosocial impacts.
Rego et al. (33)	COVID-19 vaccination refusal trends in Kenya over 2021	Kenya	Longitudinal rapid response phone surveys	11,569 (5,432)	Household cohort survey representative of the Kenyan population including refugees	40 (14) years	February and October 2021	Vaccination refusal	Factors associated with vaccination refusal	Vaccination refusal was associated with having education beyond the primary level and believing in misinformation
Orangi et al. (34)	Assessing the Level and Determinants of COVID-19 Vaccine Confidence in Kenya	Kenya	Cross-sectional study	4,136 (1355)	Participants were sampled from households in four existing Population Council prospective cohort studies across four counties: Kilifi, Kisumu, Nairobi and Wajir.	40.8 (12.6) years	February 2021	Determinants of vaccine hesitancy	Factors promoting unwillingness to receive COVID-19 vaccine.	Factors associated with vaccine hesitancy included: Rural regions, perceived difficulty in adhering to government regulations on COVID-19 prevention, no perceived COVID-19 infection risk, concerns regarding vaccine safety and effectiveness, and religious and cultural reasons.
Bono et al. (35)	Factors Affecting COVID-19 Vaccine Acceptance: An International Survey among Lowand Middle-Income Countries	Uganda	Descriptive cross-sectional study	107 (55)	Individuals 18 years and older who provided informed consent to participate in this study.	33.79 (8.84)years	10 December 2020 to 9 February 2021	Vaccine refusal	Factors associated with vaccine refusal	The main reasons underpinning vaccine refusal were fear of side effects (41.2%) and lack of confidence in vaccine effectiveness (15.1%).
Kanyanda et al. (36)	Acceptance of COVID-19 vaccines in sub-Saharan Africa: evidence from six national phone surveys	Uganda	Longitudinal high-frequency phone surveys	2,129	Respondents of national high-frequency phone surveys, aged 15 years and older, drawn from a nationally representative sample of households	≥ 15 years	December 2020	Vaccine hesitancy	Factors causing vaccine reluctance	Safety concerns about the vaccine in general and its side effects specifically emerge as the primary reservations toward a COVID-19 vaccine across countries.
Kanyike et al. (37)	Acceptance of the coronavirus disease2019 vaccine among medical students in Uganda	Uganda	Online, descriptive, cross-sectional study using a quantitative approach	600 (377)	Medical students pursuing undergraduate degree programs of choice.	≥ 18 years	Monday 15 March and Sunday 21 March 2021	Factors preventing vaccine uptake	Barriers to vaccine uptake	The most cited reasons for not taking up the vaccine were concerns about safety and having heard or read negative information about the vaccine.
Wafula et al. (38)	Intention to vaccinate against COVID-19 and adherence to non-pharmaceutical interventions against COVID-19 prior to the second wave of the pandemic in Uganda: a cross-sectional study	Uganda	Nationwide cross-sectional survey	1,053 (651)	Adults 18 years and older with access to cell phones and who had been residents in the study district for at least 6 months.	34 (18–80) years	March 2021	Reason for reluctance to get vaccinated	Barriers to vaccine uptake	Concerns for side effects were negatively associated with vaccination intent
Osur et al. (39)	Determinants of COVID-19 vaccine behavior intentions among the youth in Kenya: a cross-sectional study	Kenya	Mixed-method study using a cross-sectional survey and focused group discussion approaches.	665 (401)	Youths aged 18–35, registered in online platforms/peer groups that included Shujaaz, Brck Moja, Aifuence, Y Act and Heroes for Change.	18–35 years	Not available	Vaccine hesitancy	Reasonsa for vaccine hesitancy	Lack of information and concerns around vaccine safety and effectiveness were main cause of COVID-19 vaccine hesitancy
Shah et al. (40)	Perceptions and Knowledge toward COVID-19 Vaccine Hesitancy among a Subpopulation of Adults in Kenya: An English Survey at Six Healthcare Facilities	Kenya	Cross-sectional survey	3,996 (1789)	The general adult public (patients and relatives) visiting the inpatient and outpatient clinics from six different healthcare facilities	33 (26.5–43.0) years	November 2021 and January 2022	Vaccine hesitancy perceptions	Barriers to vaccine hesitance	Some participants reported being hesitant to take the vaccine due to side effects associated with the vaccine.

COVID-19, coronavirus disease 19; LMICs, Low—and Middle-Income Countries; WASH, water, sanitation, and hygiene; SARS-CoV-2, severe acute respiratory syndrome coronavirus-2.

### Quality appraisal

We assessed for potential bias in eligible studies using the Quality Assessment Tool for Observational Cohort and Cross-sectional studies (41). This checklist judges the quality of reporting in cohort and cross-sectional studies by evaluating aspects such as the article’s objectives, the study population, exposure measures and potential confounders, among others.

### Data synthesis and analysis

Qualitative synthesis of the main findings of the relevant articles was conducted due to the large diversity in the study designs and populations of the eligible studies. The findings were grouped based on their specific outcome, either enablers or barriers to COVID-19 vaccine uptake and handwashing practices. Further, we used tables to depict a summary of the characteristics and findings of the eligible studies.

## Results

### Study selection

**Figure 1:**
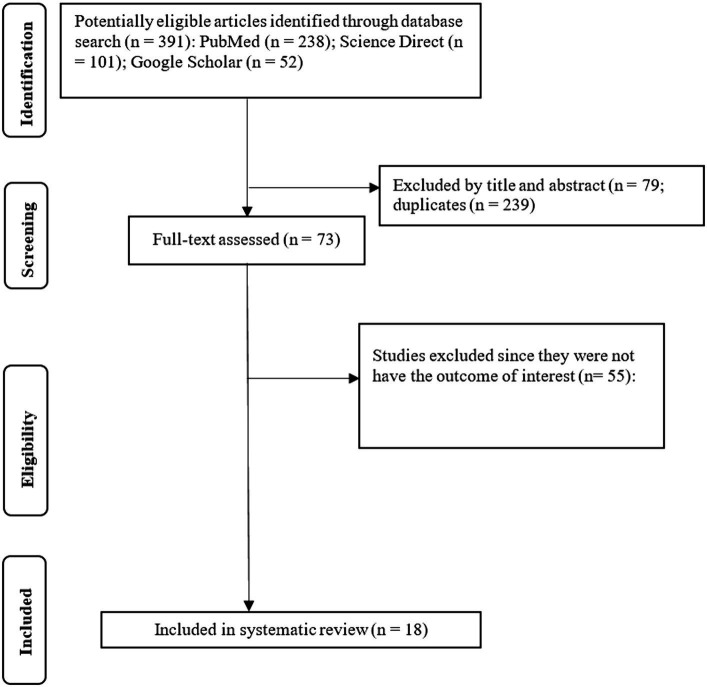
PRISMA chart depicting study selection process.

We retrieved 391 papers from our initial search of three databases, namely PubMed, Science Direct, and Google Scholar (Figure 1). Of these research articles, 239 were identified as duplicates, with 79 papers being excluded since they were irrelevant after screening by title and abstract. The remaining 73 studies underwent full-text screening, with 55 articles being excluded due to lack of the outcome of interest. Our study included a review of 18 research papers focusing on the enablers and barriers to COVID-19 vaccine uptake and handwashing practices in Kenya, Uganda and Tanzania.

### Study characteristics and outcomes

This systematic review includes 18 observation studies with publication years ranging from 2019 to 2023. Eleven studies with 18,794 participants focused on enablers of COVID-19 vaccine uptake and handwashing practice (Table 2). On the other hand, 15 of the eligible studies comprising 27,252 participants and 622 vaccination sites reported on barriers to vaccine uptake and handwashing practices (Table 1). Of note, some of the eligible studies assessed both outcomes of interest. The study designs consisted of cross-sectional studies, a qualitative case study, a mixed-methods study and a longitudinal rapid response study.

**Table 2:** Studies that reported enablers of COVID-19 vaccine uptake and handwashing practice to prevent and control COVID-19.

References	Title of paper	Country	Study design	Sample size (male)	Participants	Age [mean(sd)]	Dates of data collection	Outcome	Outcome definition	Main findings
Konje et al. (26)	The Coverage and Acceptance Spectrum of COVID-19 Vaccines among Healthcare Professionals in Western Tanzania: What Can We Learn from This Pandemic?	Tanzania	Cross-sectional study	811 (423)	Healthcare professionals of different cadres from health facilities in western Tanzania	35 (9.0) years	13 and 26 September 2021	Enablers to COVID-19 vaccine uptake	Cues for actions on improving COVID-19 vaccine uptake among health professionals	The key cues that were supported by almost half of health professionals include availability and provision of information, social support, and involvement of influential leaders during the advocacy campaign to improve COVID-19 vaccine uptake. A majority of participants reported that engagement of government authority for the provision of vaccine information, involvement of public figures in advocacy of the vaccine, and support from close family members and friends would improve the vaccine’s uptake.
Mwai et al. (27)	Assessment of water, sanitation and hygiene practices for prevention and control of COVID-19 in Kenya	Kenya	Cross-sectional survey	612 (181)	Household heads (men and women), residing in Kilifi and Mombasa counties	38.2 (14.8) years	25 November and 3 December 2020,	Enablers to handwashing	Factors that promoted handwashing and factors that hindered handwashing practices	The housed holds indicated that the information received was on water use (53.6%), hygiene (42.6%), hand washing (39.2%) and the use of soap (33.3%)
Mghamba et al. (42)	Compliance to infection prevention and control interventions for slowing down COVID-19 in early phase of disease transmission in Dar es Salaam, Tanzania	Tanzania	Cross-sectional study	390 (195)	Community members in business areas, bars and bus stands	34.8 (11.2) years	April and May 2020	Enablers of handwashing practices	Proportion of people who have received information about hand hygiene	98.4% of the respondents reported to have been informed on how to effectively wash their hands using water and soap or alcohol-based sanitizers
Macharia et al. (43)	An empirical assessment of the factors influencing acceptance of COVID-19 vaccine uptake between Kenyan and Hungarian residing populations: A cross-sectional study	Kenya and other country	Cross sectional study	1,528 (NA)	Participants currently residing in Kenya having adopted the COVID-19, WHO guidelines and protocols at an early stage of the pandemic	31.9 (9.3) years	April to August 2021	Community public awareness	Proportion of participants receiving public awareness at community level	63.7% of the participants confirmed to have received any form of public awareness at the community level, regarding the importance of the newly developed vaccines against the SARS-CoV-2 virus.
Kabagenyi et al. (29)	Factors Associated with COVID-19 Vaccine Hesitancy in Uganda: A Population-Based Cross-Sectional Survey	Uganda	Population-Based Cross-Sectional Survey	1,042 (462)	Adults ages 18 and above from rural and urban settings	40 (NA) years	June to November 2021	Demographic and socio-economic factors and COVID-19 awareness factors	Age, gender, place of residence and household size of the study participants.	Odds of COVID-19 vaccine hesitancy reduced as: education level increased, access to more sources of information, as well as having knowledge on the ways of transmitting the virus. Relatedly, male sex were less likely to be hesitant.
Abu and Elliott (32)	The critical need for WASH in emergency preparedness in health settings, the case of COVID-19 pandemic in Kisumu Kenya	Kenya	Qualitative case study design	15 (10)	County government officials and eight were NGO officials	40 (14) years	August and September 2020	Vaccine and Handwashing practices	Level of preparedness of accessing handwashing	All participants indicated the healthcare system was ill-prepared for the pandemic. Health care workers experienced such severe psychosocial impacts due to the lack of preparedness that they subsequently embarked on strikes in protest.
Rego et al. (33)	COVID-19 vaccination refusal trends in Kenya over 2021	Kenya	Longitudinal rapid response phone surveys	11,569 (5,432)	Household cohort survey representative of the Kenyan population including refugees		February and October 2021	Reduction in vaccination refusal	Factors associated with decreased vaccination refusal	Having an education beyond the primary level was associated with a 4.1[0.7,8.9] reduction in vaccination refusal.
Bono et al. (35)	Factors Affecting COVID-19 Vaccine Acceptance: An International Survey among Lowand Middle-Income Countries	Uganda	Descriptive cross-sectional study	107 (55)	Individuals 18 years and older who provided informed consent to participate in this study.	33.79 (8.84)years	10 December 2020 to 9 February 2021	Vaccine acceptance`	Factors associated with vaccine acceptance	Vaccine acceptance was positively associated with COVID-19 knowledge, worry/fear regarding COVID-19, higher income, younger age, and testing negative for COVID-19.
Echoru et al. (44)	Sociodemographic factors associated with acceptance of COVID-19 vaccine and clinical trials in Uganda: a cross-sectional study in western Uganda	Uganda	Cross-sectional study	1,067 (781)	Adults of 18 to 70 years of age who had smartphones, and were capable of reading or using the Internet.	18–70 years	July to September 2020	Vaccine acceptance	Promoters of vaccine acceptancy	Those who ended at the tertiary level of education and students were more likely to accept the vaccine (OR: 2.8; 95%CI: 1.25–6.11; P = 0.009).
Kanyike et al. (37)	Acceptance of the coronavirus disease2019 vaccine among medical students in Uganda	Uganda	Online, descriptive, cross-sectional study using a quantitative approach	600 (377)	Medical students pursuing undergraduate degree programs of choice.	≥ 18 years	Monday 15 March and Sunday 21 March 2021	Factors promoting vaccine uptake	Enablers to vaccine uptake	The major reasons for acceptance were to protect oneself (n = 191, 85.3%) and others (n = 142, 63.4%) from COVID-19.
Wafula et al. (38)	Intention to vaccinate against COVID-19 and adherence to non-pharmaceutical interventions against COVID-19 prior to the second wave of the pandemic in Uganda: a cross-sectional study	Uganda	Nationwide cross-sectional survey	1,053 (651)	Adults 18 years and older with access to cell phones and who had been residents in the study district for at least 6 months.	34 (18–80) years	March 2021	Reasons for intending to get vaccinated	Enablers to vaccine uptake	Concerns about the chances of getting COVID-19 in the future and fear of severe COVID-19 infection were the strongest predictors for a definite intention

COVID-19, coronavirus disease 19; NGO, non-governmental organization; SARS-CoV-2, severe acute respiratory syndrome coronavirus-2.

### Quality evaluation

According to the Quality Assessment Tool for Observational Cohort and Cross-sectional Studies checklist, all the studies met the recommendations for conducting observational studies (Supplementary Table S3). This finding indicates high overall methodological quality and relatively low risk of bias of these studies.

### Narrative synthesis

#### Enablers to COVID-19 vaccine uptake

##### Provision of information and social support on COVID-19 vaccines

Health professionals participating in a study in Tanzania indicated that the availability and provision of information promoted COVID-19 vaccine uptake (26). Similarly, Macharia and colleagues reported that 63.7% of study participants agreed to have received public awareness at the community level regarding the importance of the newly developed vaccines against the SARS-CoV-2 virus (43). Access to more sources of information as well as having knowledge on the ways of transmitting the virus promoted COVID-19 vaccine uptake (29).

ss93% of respondents reported awareness of the COVID-19 pandemic at the community level, while 47% showed awareness of the COVID-19 pandemic at the county health system level (32). Acceptance of the COVID-19 vaccine had a positive association with COVID-19 knowledge and the need to protect oneself and others from infection with SARS-CoV-2 (35, 37). Participants in a study understood that vaccination with the COVID-19 vaccine would protect other people in the community (39). Of note, knowledge and availability of social support on vaccine uptake empower individuals to take the vaccine and to take effective preventive measures.

##### Level of education

A longitudinal rapid response phone survey indicated that vaccine refusal was associated with a dramatic decline throughout 2021, from 24% in February 2021 to 9% in October 2021 (45). In this case, individuals with education beyond the primary level had low levels of vaccine hesitancy (45). A Ugandan study of adults from rural and urban settings reported that an increased level of education reduced the odds of COVID-19 vaccine hesitancy (29). According to Echoru and colleagues, individuals who ended at the tertiary level of education and students were more likely to accept the vaccine (44). High education levels probably increases the understanding of the need to be vaccinated, hence increasing vaccine acceptance among individuals.

##### Fear of severe infection

Another motivator for accepting the COVID-19 vaccine is the fear of getting SARS-CoV-2 infection and subsequent development of severe COVID-19, as reported by Wafula et al. (38).

##### Involvement of influential leaders

A cross-sectional study by Konje et al. (26) showed that the involvement of community and religious leaders during the advocacy campaign improved COVID-19 vaccine uptake among health professionals. Moreover, a majority of participants reported that engagement of government authority in the provision of vaccine information, involvement of public figures in advocacy of the vaccine, and support from close family members and friends would improve the vaccine’s uptake (26).

#### Enablers of handwashing practices

##### Provision of information on hand hygiene

A cross-sectional survey involving household heads from Kilifi and Mombasa counties in Kenya reported that 53.6% of the households had received information on water use, while 42.6% had information on hygiene, 39.2% on handwashing and 33.3% on the use of soap. The information most received by households on water and sanitation focused on handwashing with soap (91.7%), the use of alcohol-based hand sanitizer (43.3%) and safe storage of household water (41.5%) (27). Mghamba et al. reported that 98.4% of the community members in business areas, bars and bus stands were informed about effective washing of their hands using water and soap or alcohol-based sanitizers (42).

##### Access to soap and water

A survey assessing water, sanitation and hygiene practices in Kenya reported that about 59% of participating households had enough water to meet their demand, with 97% indicating that they practice handwashing (27). Similarly, proper handwashing practices were observed in areas with low populations, particularly in supermarkets that provided soap and water (42).

#### Barriers to uptake of vaccines in Kenya, Uganda, and Tanzania

##### Lack of access to vaccines

A study focusing on Kenya indicated that an increase in mean travel times to vaccination sites was associated with a decreased probability of COVID-19 vaccine uptake. Additionally, there was a negative association between vaccination coverage and the proportion of the population residing in rural areas, with a 27.8% decline (28).

##### Awareness barriers

High levels of vaccine hesitancy, 58.6%, in Uganda were associated with inadequate awareness of vaccination sites and vaccine types (29). A mixed-method study indicated that lack of information on the COVID-19 vaccine was the main barrier to vaccine uptake (39).

##### Fear of side effects and lack of trust in the COVID-19 vaccine

Concerns about side effects of the COVID-19 vaccine among respondents and distrust in vaccine stakeholders were the key barriers to vaccine uptake in Uganda (30). In another setting, the experiences of key informants in Kenya noted that a lack of trust in the information regarding the efficacy and safety of the COVID-19 vaccines provided by the health authorities reduced vaccine uptake (32). Lack of confidence in COVID-19 vaccine effectiveness and safety concerns were major concerns preventing participants from taking the COVID-19 vaccine (31, 35–40).

##### Myths and misconceptions

Adult respondents from rural and urban settings in Uganda were hesitant to receive the COVID-19 vaccine due to myths and misconceptions about the SARS-CoV-2 virus and the vaccine itself (29). For instance, 15% of the participants believed that the vaccine could cause infertility or that the vaccine could infect them by spreading the virus into their bodies. Other reasons hindering vaccine uptake are the beliefs that COVID-19 is not a serious illness and kills only people with underlying medical conditions (29). Moreover, religious and cultural beliefs hindered COVID-19 vaccine uptake (33).

#### Barriers to handwashing practices in Kenya, Uganda, and Tanzania

##### Lack of access to water and soap

Lack of access to water and the likelihood of paying for water was a challenge to the uptake of handwashing in Kenya (27). Similarly, it was noted that inadequate access to hygiene and safety caused psychosocial stresses among healthcare workers, thus affecting the quality of care provided (32).

##### Inadequate awareness

Assessment of the knowledge about hand hygiene practices during the COVID-19 pandemic among residents in Mombasa and Kilifi counties in Kenya found that the information received on hygiene practices was below 50% (27). In particular, this article showed that access to information about hygiene among the participating households was 42.6%, handwashing and the use of soap being 39.2 and 33.3%, respectively.

##### Mistrust of the governments’ messages on hand hygiene

Individuals did not trust the government’s recommendations or messaging on the benefits of hand hygiene, hence reducing handwashing practices (32).

## Discussion

This systematic review aimed to synthesize evidence on enablers and barriers to vaccine uptake and handwashing practice in Kenya, Uganda, and Tanzania to prevent and control COVID-19. Our results acknowledge that access to vaccines, hand hygiene programs and availability of water are crucial in promoting vaccine uptake and handwashing behavior among individuals. On the contrary, lack of awareness about vaccines and handwashing facilities, inaccessibility of vaccines and handwashing resources hinder the effective use of the vaccine and washing of hands.

Our findings are similar to previous studies, which indicated that providing facilities with clean water, soap, basins, and hand rub increased handwashing practice (45). Similarly, previous research on access to knowledge and information about hand hygiene and COVID-19 vaccines has presented similar findings (11). The author’s findings revealed that participants who had received formal hand hygiene training applied the practice routinely during the COVID-19 pandemic. Our results are consistent with the conclusion by Alegbeleye and colleagues that improving hand hygiene practices requires providing education on hand hygiene practices (12). These findings emphasize the importance of community leaders’ involvement as supported by previous research showing that collaboration between community leaders and governments improved the rates of vaccination (14). In addition, community involvement in vaccine delivery has been found to increase vaccine uptake (15). This may be attributed to their familiarity with the community dynamics and ability to communicate effectively in the local language, a critical factor in rural areas where COVID-19 vaccine information must be conveyed in the native tongue. This challenge was effectively addressed by capitalizing on existing community structures, such as baraza gatherings, which are deeply ingrained in the East African context (46). These gatherings serve as invaluable platforms for sensitizing rural communities in their local dialects about the importance of COVID-19 vaccines. This approach is corroborated by qualitative research findings, where a rural community in a high-income country adopted a multifaceted approach. This included translating vaccine information into the local dialect on posters, leveraging local radio broadcasts, and mobilizing community champions to bolster vaccine confidence (47).

In concordance with our findings, studies have shown that access to knowledge and information about the importance of vaccination, the benefits, the side effects, and the risks have helped people to make informed vaccination decisions (48, 49). In particular, Bongomin et al. reported that 70.1% of respondents were willing to be vaccinated due to increased sensitization. A review focusing on low and middle-income countries (LMICs), including Uganda, noted that due to awareness focusing on topics such as vaccine efficacy and safety (49).

Regarding barriers to the COVID-19 vaccine uptake and handwashing practice, the study findings agree with previously published evidence. Assessment of hand hygiene barriers in a teaching hospital’s intensive care unit in southeast Iran found that lack of quality equipment reduced hand hygiene practices (50). Further, insufficient quality equipment as evidenced by poor quality soap was associated with skin dryness and itching and a shortage of disinfectants led to minimal hand hygiene compliance (50). Another study in Indonesia revealed that limited water led to poor handwashing practices (10). Additionally, our findings align with a review that identified infrastructural deficits, such as a lack of water and soap, as barriers to hand hygiene practices (51). Notably, our synthesis resonates with findings from Naidoo’s review, which highlighted widespread fears over potential side effects and concerns about the newly developed COVID-19 vaccine being perceived as unsafe for the African population (17). Similarly, our results parallel Naidoo’s observation that a substantial number of studies across different contexts expressed concerns regarding the vaccine’s perceived ineffectiveness in providing protection against COVID-19 (17).

Safety concerns, misinformation, and a lack of trust in the government have influenced the low uptake of vaccines. Previous research has reported similar findings. Rutten and colleagues highlighted that a lack of trust in COVID-19 vaccines resulted in vaccine reluctance (14). Similarly, the rapid pace of vaccine development and rampant misinformation in social media has hindered successful vaccine uptake. A study exploring COVID-19 vaccine hesitancy revealed that fear of needles or blood, safety concerns, costs, and religious beliefs had led to low vaccine uptake (16).

In addition to the distinctive factors highlighted in our narrative synthesis regarding vaccine uptake and handwashing practices in East African countries, it is noteworthy to draw parallels with results from high-income countries, such as Portugal. Low confidence in the COVID-19 vaccines being developed and the perception that the information provided by health authorities during the pandemic was inconsistent and contradictory were identified as barriers to vaccine uptake (52), potentially fueled by the rapid development of COVID-19 vaccines amid the urgency of the pandemic. However, it is crucial to acknowledge that high-income countries often demonstrated a higher level of preparedness in terms of healthcare infrastructure and vaccine programs, compared to lower-middle-income countries (LMICs) like those in East Africa.

Our narrative synthesis highlights the challenges faced by LMICs, including limited funding for COVID-19 vaccine programs and hand hygiene practices, which posed significant barriers. Unlike high-income countries with more extensive resources, LMICs grappled with constrained healthcare budgets and infrastructural deficits, amplifying the difficulties in implementing effective health interventions. Recognizing these disparities prompts a call for more extensive and meticulous post-pandemic research to comprehensively understand our systems’ complexities during the crisis. Such research will be instrumental in identifying the flaws in our preparedness, informing targeted strategies for future health crises, and ensuring the equitable distribution of resources for effective public health interventions. Furthermore, we advocate for future interventions that prioritize education and early engagement of local community leaders. Tailoring educational campaigns to the socio-cultural contexts of Kenya, Uganda, and Tanzania and involving community leaders from the outset is crucial for enhancing the effectiveness of initiatives promoting vaccine uptake and handwashing practices. Proactive community involvement fosters trust, heightens awareness, and contributes to the successful adoption of health practices.

Our study is the first systematic review focusing on the enablers and barriers to COVID-19 vaccine uptake and handwashing to prevent and control COVID-19 in East Africa. This review can be used as a baseline for similar research within this region. However, we acknowledge that the study was limited to three nations; hence our findings may not be generalizable to other regions due to varying political, environmental, economic, and social factors. Additionally, we acknowledge limitations in the search for relevant literature due to accessibility issues, noting the search was limited to three databases: PubMed, ScienceDirect, and Google Scholar. PubMed was chosen for its comprehensive coverage of biomedical literature, including COVID-19-related research (53, 54). ScienceDirect offers diverse scientific journals (55), while Google Scholar enhances comprehensiveness by covering both peer-reviewed and non-peer-reviewed sources. Despite valuable databases like Web of Science and Scopus being inaccessible due to subscription requirements, the focus on freely accessible databases aimed to optimize efficiency and transparency. While acknowledging the potential for missed literature, the team mitigated this by rigorously screening studies based on predefined criteria.

## Conclusion

This study contributes to the understanding of the factors affecting COVID-19 vaccine uptake and handwashing behavior in Kenya, Uganda and Tanzania. Among the enablers of vaccine uptake and handwashing practice are the accessibility of vaccines. soap and water, and the involvement of local leaders. Conversely, lack of awareness and fear of side effects hinders individuals from taking the COVID-19 vaccine and practicing effective handwashing. Our systematic review points to the need for a more extensive scope of research to increase the generalizability of the study findings.

## Data availability statement

The original contributions presented in the study are included in the article/Supplementary material, further inquiries can be directed to the corresponding author.

## Author contributions

JMM: Conceptualization, Data curation, Formal analysis, Investigation, Methodology, Project administration, Supervision, Validation, Visualization, Writing – original draft, Writing – review & editing. JM: Methodology, Validation, Visualization, Writing – review & editing. HM: Data curation, Methodology, Writing – review & editing. KK: Data curation, Methodology, Writing – review & editing. SK: Data curation, Methodology, Writing – review & editing. LK: Data curation, Methodology, Writing – review & editing. RM: Data curation, Methodology, Writing – review & editing. MN: Data curation, Writing – review & editing.
