# Supplementary material for: Enablers and barriers to vaccine uptake and handwashing practices to prevent and control COVID-19 in Kenya, Uganda, and Tanzania: a systematic review
Source: Front Public Health. 2024 Mar 27;12:1352787. doi: 10.3389/fpubh.2024.1352787 (PMC11004251; doi:10.3389/fpubh.2024.1352787)
Supplement: Supplementary file 1 [file Table_1.DOCX]

# Supplementary Tables

| Supplementary table 1. Search terms and their keywords used to formulate medical subject heading (MeSH) terms. | | |
| --- | --- | --- |
| **Term** | **Key words** | **MeSH terms** |
| Handwashing | Handwashing  Hand hygiene | ("hand disinfection"[MeSH Terms] OR Handwashing[Text Word] OR "hand hygiene"[MeSH Terms] OR hand hygiene[Text Word]) |
| Vaccination | Vaccine  Vaccination  Immunization | ("vaccination"[MeSH Terms] OR vaccination[Text Word] OR "immunization"[MeSH Terms] OR immunization[Text Word]) |
| COVID-19 | COVID-19  SARS-CoV2 | ("COVID-19"[All Fields] OR "COVID-19"[MeSH Terms] OR "SARS-CoV-2"[All Fields] OR "sars-cov-2"[MeSH Terms] OR "Severe Acute Respiratory Syndrome Coronavirus 2"[All Fields]) |
| Prevention | Prevention  Control | ("prevention and control"[Subheading] OR prevention[Text Word] OR "control groups"[MeSH Terms] OR control[Text Word]) |
| Enablers | Enablers | Enabler[Text Word] |
| Barriers | Barriers | Barrier[Text Word] |
| Policy | Policy | ("policy"[MeSH Terms] OR policy[Text Word]) |
| Kenya | Kenya | ("Kenya"[MeSH Terms] OR Kenya[Text Word]) |
| Uganda | Uganda | ("Uganda"[MeSH Terms] OR Uganda[Text Word]) |
| Tanzania | Tanzania | ("Tanzania"[MeSH Terms] OR Tanzania[Text Word]) |
| **Abbreviations and acronyms:** COVID-19, Coronavirus disease 2019; SARS-CoV2, severe acute respiratory syndrome coronavirus 2; MeSH, Medical subject heading.  We derived the keywords from the research question and used them to identify MeSH terms on PubMed database. | | |

| Supplementary table 2. Search strategy used to identify the relevant papers published on PubMed database. | | | |
| --- | --- | --- | --- |
| **Database** | **Date searched** |  | **Search strategy** |
| **PubMed** | 06/11/2023 | (((term 1) OR (term 2)) AND ((term 3) AND (term 4)) AND ((term 5) OR (term 6)) AND (term 7) AND ((term 8) OR (term 9) OR (term 10))) | ((("hand disinfection"[MeSH Terms] OR Handwashing[Text Word] OR "hand hygiene"[MeSH Terms] OR hand hygiene[Text Word]) OR ("vaccination"[MeSH Terms] OR vaccination[Text Word] OR "immunization"[MeSH Terms] OR immunization[Text Word])) AND (("COVID-19"[All Fields] OR "COVID-19"[MeSH Terms] OR "SARS-CoV-2"[All Fields] OR "sars-cov-2"[MeSH Terms] OR "Severe Acute Respiratory Syndrome Coronavirus 2"[All Fields]) AND ("prevention and control"[Subheading] OR prevention[Text Word] OR "control groups"[MeSH Terms] OR control[Text Word])) AND ((Enabler[Text Word]) OR (Barrier[Text Word])) AND ("policy"[MeSH Terms] OR policy[Text Word]) AND (("Kenya"[MeSH Terms] OR Kenya[Text Word]) OR ("Uganda"[MeSH Terms] OR Uganda[Text Word]) OR ("Tanzania"[MeSH Terms] OR Tanzania[Text Word]))) |
| **Google scholar** | 06/11/2023 | (((term 1) OR (term 2)) AND ((term 3) AND (term 4)) AND ((term 5) OR (term 6)) AND (term 7) AND ((term 8) OR (term 9) OR (term 10))) | (((Handwashing OR hand hygiene) OR (vaccination OR immunization)) AND ((“COVID-19" OR "SARS-CoV-2" OR "Severe Acute Respiratory Syndrome Coronavirus 2") AND ("prevention and control" OR prevention OR control)) AND (( Enabler) OR ( Barrier)) AND (policy) AND ((Kenya) OR (Uganda) OR (Tanzania))) |
| **Science Direct** | 06/11/2023 | (((term 1) OR (term 2)) AND ((term 3) AND (term 4)) AND ((term 5) OR (term 6)) AND (term 7) AND ((term 8) OR (term 9) OR (term 10))) | (((Handwashing OR hand hygiene) OR (vaccination OR immunization)) AND ((“COVID-19" OR "SARS-CoV-2" OR "Severe Acute Respiratory Syndrome Coronavirus 2") AND ("prevention and control" OR prevention OR control)) AND (( Enabler) OR ( Barrier)) AND (policy) AND ((Kenya) OR (Uganda) OR (Tanzania))) |
| **Abbreviations and acronyms:** COVID-19, Coronavirus disease 2019; SARS-CoV2, severe acute respiratory syndrome coronavirus 2; MeSH, Medical subject heading.  The search strategy was created by merging the keywords: handwashing, vaccine, COVID-19, prevention, policy, barriers, enablers, Kenya, Uganda, and Tanzania, which was later customized to suit various databases. | | | |

Supplementary table 3. Quality Assessment Tool for Observational Cohort and Cross-sectional studies

|  | **Mghamba, 2022** | **Kanyanda, 2021** | **Echoru, 2021** | **Bono, 2021** | **Kanyike, 2021** | **Wafula, 2022** | **Ouni, 2023** | **Mwai, 2022** | **Osur, 2022** | **Macharia, 2022** | **Orangi, 2021** | **Muchiri, 2022** | **Shah, 2022** | **Abu 2022** | **Kabagenyi, 2022** | **Konje, 2022** | **Ocholla, 2021** | **Rego, 2021** |
| --- | --- | --- | --- | --- | --- | --- | --- | --- | --- | --- | --- | --- | --- | --- | --- | --- | --- | --- |
| Was the research question or objective clearly stated? | YES | YES | YES | YES | YES | YES | YES | YES | YES | YES | YES | YES | YES | YES | YES | YES | YES | YES |
| Was the study population clearly specified and defined? | YES | YES | YES | YES | YES | YES | YES | YES | YES | YES | YES | YES | YES | YES | YES | YES | YES | YES |
| Was the participation rate of eligible persons at least 50%? | YES | YES | YES | YES | YES | YES | YES | YES | YES | YES | YES | YES | YES | YES | YES | YES | YES | YES |
| Were all the subjects selected or recruited from the same or similar populations (including the same time period)? Were inclusion and exclusion criteria for being in the study pre-specified and applied uniformly to all participants? | YES | YES | YES | YES | YES | YES | YES | YES | YES | YES | YES | YES | YES | YES | YES | YES | YES | YES |
| Was a sample size justification, power description, or variance and effect estimates provided? | YES | YES | YES | NO | YES | YES | YES | YES | YES | YES | YES | YES | NO | NO | YES | YES | NO | NO |
| For the analyses in this paper, were the exposure(s) of interest measured prior to the outcome(s) being measured? | YES | YES | YES | YES | YES | YES | YES | YES | YES | YES | YES | YES | YES | YES | YES | YES | YES | YES |
| Was the timeframe sufficient so that one could reasonably expect to see an association between exposure and outcome if it existed? | YES | YES | YES | YES | YES | YES | YES | YES | YES | YES | YES | YES | YES | YES | YES | YES | YES | YES |
| For exposures that can vary in amount or level, did the study examine different levels of the exposure as related to the outcome (e.g., categories of exposure, or exposure measured as continuous variable)? | NA | NA | NA | NA | NA | NA | NA | NA | NA | NA | NA | NA | NA | YES | YES | NA | NA | ,YES |
| Were the exposure measures (independent variables) clearly defined, valid, reliable, and implemented consistently across all study participants? | YES | YES | YES | YES | YES | YES | YES | YES | YES | YES | YES | YES | YES | YES | YES | YES | YES | YES |
| Was the exposure(s) assessed more than once over time? | NA | NA | NA | NA | NA | NA | NA | NA | NA | NA | NA | NA | NA | NA | NA | NA | NA | NA |
| Were the outcome measures (dependent variables) clearly defined, valid, reliable, and implemented consistently across all study participants? | YES | YES | YES | YES | YES | YES | YES | YES | YES | YES | YES | YES | YES | YES | YES | YES | YES | YES |
| Were the outcome assessors blinded to the exposure status of participants? | NA | NA | NA | NA | NA | NA | NA | NA | NA | NA | NA | NA | NA | NA | NA | NA | NA | NA |
| Was loss to follow-up after baseline 20% or less? | NA | NA | NA | NA | NA | NA | NA | NA | NA | NA | NA | NA | NA | NA | NA | NA | NA | NA |
| Were key potential confounding variables measured and adjusted statistically for their impact on the relationship between exposure(s) and outcome(s)? | NR | NR | NR | NR | NR | YES | NR | NR | NR | NR | NR | NR | NR | NR | YES | NR | NR | NR |
| Quality score (%) | 90 | 90 | 90 | 80 | 90 | 100 | 90 | 90 | 90 | 90 | 90 | 90 | 80 | 82 | 100 | 90 | 80 | 82 |
|  |  |  |  |  |  |  |  |  |  |  |  |  |  |  |  |  |  |  |
|  |  |  |  |  |  |  |  |  |  |  |  |  |  |  |  |  |  |  |
| *CD, cannot determine; NA, non applicable; NR, not registered |  |  |  |  |  |  |  |  |  |  |  |  |  |  |  |  |  |  |
|  |  |  |  |  |  |  |  |  |  |  |  |  |  |  |  |  |  |  |
